# Measles–Rubella Microarray Patches Phase III Clinical Trial Framework: Proposal and Considerations

**DOI:** 10.3390/vaccines12111258

**Published:** 2024-11-06

**Authors:** Darin Zehrung, Bruce L. Innis, Auliya A. Suwantika, Mahmoud Ameri, Robin Biellik, James C. Birchall, Alejandro Cravioto, Courtney Jarrahian, Lee Fairlie, James L. Goodson, Sonali Kochhar, Katrina Kretsinger, Christopher Morgan, Mercy Mvundura, Niraj Rathi, Edward Clarke, Jessica Joyce Mistilis, Marie-Chantal Uwamwezi, Birgitte Giersing, Mateusz Hasso-Agopsowicz

**Affiliations:** 1 World Health Organization, 1211 Geneva, Switzerland; 2 Independent Researcher, Haverford, PA 19041, USA; 3 Department of Pharmacology and Clinical Pharmacy, Faculty of Pharmacy, Universitas Padjadjaran, Bandung 45363, Indonesia; 4 Independent Researcher, Fremont, CA 9453, USA; 5 Independent Researcher, 1278 La Rippe, Switzerland; 6 School of Pharmacy and Pharmaceutical Sciences, Cardiff University, Cardiff CF10 3NT, UK; 7 Departamento de Salud Pública, Facultad de Medicina, National Autonomous University of Mexico, Mexico City 04510, Mexico; 8 PATH, Seattle, WA 98121, USA; 9 Wits RHI, University of Witwatersrand, Johannesburg 2001, South Africa; 10 Centers for Disease Control and Prevention, Atlanta, GA 30333, USA; 11 Department of Global Health, University of Washington, Seattle, WA 98105, USA; 12 Independent Researcher, Decatur, GA 30033, USA; 13 JHPIEGO, Baltimore, MD 21231, USA; 14 PATH, New Delhi 110001, India; 15 Medical Research Council Unit, The Gambia at the London School of Hygiene and Tropical Medicine, Banjul P.O. Box 273, The Gambia; 16 PATH, 1040 Brussels, Belgium

**Keywords:** measles, rubella, vaccines, micropatch, clinical trial

## Abstract

**Background**: The Measles–Rubella Microarray Patch (MR-MAP) is an important technology that is expected to reduce coverage and equity gaps for measles-containing vaccines (MCVs), reach zero-dose children, and contribute to elimination of measles and rubella. MR-MAPs are anticipated to be easier to deploy programmatically and could be delivered by lesser-trained health workers, thereby increasing immunization coverage. The most advanced MR-MAP has reached clinical proof-of-concept through a Phase I/II trial in the target population of infants and young children. The World Health Organization (WHO) and partners have developed the Phase III clinical trial framework for MR-MAPs presented in this article. **Objectives and Methods**: The purpose of such framework is to inform the considerations, design and approach for the pivotal clinical trial design, while considering the anticipated data requirements to inform regulatory approval, WHO prequalification, and policy decision. **Results**: The proposed Phase III trial would compare the immunogenicity and safety of an MR-MAP with MR vaccine delivered subcutaneously in 9- to 10-month-old infants. An analysis of non-inferiority (NI) of immunogenicity would occur six weeks after the first dose. Should regulatory agencies or policy makers require, a proportion of infants could receive a second dose of either the same or alternate MR vaccine presentation six months after the first dose, with those children returning six weeks after the second dose for a descriptive assessment of immunogenicity, and then followed up six months after the second dose for evaluation of safety and immunogenicity. It is anticipated that this proposed pivotal Phase III trial framework would generate the required clinical data for regulatory licensure and WHO prequalification (PQ) of MR-MAPs. However, the trial design would need to be reviewed and confirmed by a national regulatory authority (NRA) that will assess the product for regulatory licensure and the WHO PQ team. Additional research will likely be required to generate data on concomitant vaccine delivery, the safety and immunogenicity of MR-MAPs in other age groups such as children 1–5 years and infants younger than 9 months of age, and the impact of MR-MAPs on coverage and equity. Such studies could be conducted during or after clinical MR-MAP development.

## 1. Introduction

Between 2021 and 2022, modeled global measles cases increased by 18% from 7,802,000 to 9,232,300, and the number of countries facing large or disruptive outbreaks rose from 22 to 37 [1]. In 2023, approximately 35 million children missed their first or second dose of measles-containing vaccine (MCV1 and MCV2), with 83% receiving MCV1 and 74% receiving both doses [2]. The WHO recommends that countries should strive to attain and sustain a minimum of 95% MCV coverage for both doses [3]. While vaccine effectiveness of a single dose rubella-containing vaccine (RCV) is higher than MCV, it is recommended that RCV in a 2-dose schedule be provided in combination with the measles vaccine, and the goal for rubella vaccination coverage should also be >95% given the common presentation for the vaccine [4]. Despite considerable progress incorporating the RCV into routine immunization programs globally, approximately 25 million infants still do not have access to the RCV every year [5]. Modeled estimates show a 66% decrease in the worldwide burden of congenital rubella syndrome (CRS) from 2010 to 2019; however, over 32,000 infants are still born with CRS annually, nearly all in countries that have not introduced a routine dose of the RCV [6].

Currently available MCVs are in a vial presentation and are lyophilized, requiring reconstitution with diluent prior to immunization use. They are reconstituted with a reuse prevention syringe and administered with an auto-disable needle and syringe (N&S). MCVs are often in a multi-dose vial presentation, which can result in vaccine wastage as any remaining doses in reconstituted open vials must be discarded within 6 h of opening. This has led to vaccinator reluctance in opening multi-dose vials, which results in missed opportunities for vaccination [7,8,9].

There are challenges with N&S-delivered MCV that constrain immunization programs from reaching high vaccine coverage. Examples include the requirements for vaccinators to reconstitute and administer a vaccine, the logistics of reaching remote populations due to limited vaccine thermostability, as well as safe sharps waste disposal [9]. 

Microarray patches (MAPs) are novel vaccine delivery devices in product development comprising multiple microprojections coated with, or composed of, a vaccine. Upon application to the skin, the vaccine is delivered to the dermis and epidermis in a usually less painful and minimally invasive manner than N&S. This single dose, ready-to-use presentation is expected to be easier to use, will not require vaccine reconstitution, nor require additional supplies such as syringes and, therefore, will reduce open-vial wastage and sharps waste, and is likely to have enhanced thermostability [10,11], when compared to vaccines delivered by N&S. Such attributes are anticipated to bring many opportunities to increase equitable vaccination coverage; for example, a possibility of administration of MR-MAPs by community health workers, immunization of hard-to-reach populations, the potential for partial distribution beyond the cold chain, and fewer missed opportunities for vaccination. The opportunities are likely to translate to public health impact—it has been estimated that between 2030 and 2040, the use of MR-MAPs in routine immunization and supplementary immunization activities (SIAs), partially replacing N&S use, could reduce measles burden by 27–37% in 70 low- and middle-income countries (LMICs) compared to the current N&S-only immunization strategy [12]. The evaluation and further validation of MR-MAP use cases in nine countries highlighted that the use of MR-MAPs in outreach immunization and SIAs would have the highest impact on MR vaccine coverage [9,13]. As such, MR-MAPs are anticipated to play a crucial role in eliminating measles and rubella and have been designated as a priority innovation by global initiatives such as Vaccine Innovation Prioritization Strategy, global stakeholders [14,15,16], and the Measles & Rubella Partnership [17].

There are two MR-MAP candidates that are currently in clinical development. A dissolving MAP from Micron Biomedical Inc has recently been evaluated in a Phase I/II trial in 15-month-old MR vaccinated toddlers and 9-month-old MR naïve infants in the Gambia. The trial reported that MR vaccines delivered by a MAP have comparable safety and immunogenicity to MR vaccines delivered by N&S [18,19]. Another candidate, a high-density vaccine-coated microarray patch (HD-MAP) developed by Vaxxas, has been evaluated in a Phase I trial in previously MR vaccinated, healthy Australian young adults. The trial reported that the MR vaccine delivered by a MAP has similar levels of safety and immunogenicity as the MR vaccine delivered by N&S [20]. Other MR-MAP candidates are in preclinical development. In June 2023, the WHO’s Product Development for Vaccines Advisory Committee (PDVAC) held a meeting to discuss the Phase I/II proof-of-concept clinical trial results presented by Micron Biomedical and provide recommendations for further clinical development and implementation research for MR-MAPs (Box 1) [21].

Box 1 **2023 PDVAC MR-MAP Recommendations [21].**

- A regulatory strategy should be developed to allow for the quickest route of product approval and WHO PQ, which would be with WHO prequalified MR vaccines.
- Priority use cases are campaigns and supplementary immunization activities and outbreak response.
- Conduct safety and immunogenicity assessment in naïve 9-month-old infants to optimize dose, wear time and anatomical site.
- Assess an application on the wrist for a 1-min wear time with the dose used in the Phase I/II study, shifting to a 5-min wear time and a different application site out of the reach of the child, depending on the data generated.
- MR-MAP product labeling for a VVM 30 (stability at 37 °C) as well as controlled temperature chain (CTC), which requires thermostability at 40 °C for a minimum of 3 days to allow for the use case in supplemental immunization activities (SIA—campaigns) and outbreak scenarios.
- Evaluate the potential for dose reduction and the possible impact on thermostability.
- In future clinical research, include mild and moderately malnourished infants in a Phase II study and severely malnourished infants in a Phase III study.
- Identify vaccines that could be co-administered with MR-MAPs and related use cases.
- Expanded use/indications for MR-MAPs: inclusion of 6-month old infants, next generation MR vaccines, HIV+ individuals.
- Develop Evidence Considerations for Vaccine Policy (ECVP) for MR-MAPs.

The first MR-MAP for use is anticipated to be available for introduction by 2030, following product design finalization, construction and validation of an initial commercial manufacturing facility, Phase III clinical testing, approval of regulatory applications for marketing authorization, and prequalification (PQ) by the WHO. The initial commercial production lines are anticipated to launch by 2030, with limited supply at product launch until manufacturing scale-up is achieved. Further manufacturing scale-up or scale-out will depend upon demand from end-users and additional investment from vaccine developers.

While the pilot-scale manufacturing facilities are being designed and built, an opportunity exists to refine the MR-MAP design, dose, wear time, and anatomical site for delivery, prepare for future programmatic implementation and to discuss the confirmatory clinical data required to support licensure for MR-MAPs.

Therefore, the purpose of this manuscript is to develop a framework to inform considerations, design, and approach for the Phase III trial for MR-MAPs. Through such framework, this manuscript helps ensure that data on MR-MAPs required to inform regulatory and policy decisions are available, limiting the delay in introducing this important innovation to the immunization programs of countries. To support such goal, the WHO, with support from partners, organized a Global Convening of measles and rubella experts, regulators, policymakers, MAP developers, and vaccine manufacturers on MR-MAPs that occurred in April 2024 in New Delhi, India. Here, we report on the background work, discussions, and recommendations and propose an approach for a pivotal MR-MAP Phase III clinical trial.

## 2. Phase III Trial Framework—Methods

The following methods and approaches were implemented to develop a Phase III trial framework that describes parameters intended to inform marketing application approval by regulatory authorities and WHO policy and prequalification requirements.

### 2.1. Desk-Based Research and Stakeholder Interviews

Between November 2023 and February 2024, the WHO team conducted a desk-based review of published literature accessed through PubMed, determined to be relevant to the design of a pivotal Phase III trial for MR-MAPs. The review also included published strategy and policy documents, guidelines, reports, and other grey literature documents (see Table 1): the WHO position papers on measles and rubella, the Technical Report Series on the requirements for measles, mumps, and rubella vaccines and combined vaccine as well as clinical trials for vaccines, the WHO/UNICEF MR-MAP Target Product Profile, the PDVAC report from the MR-MAP meeting held June 2023, past published research on MCV and alternative delivery technologies, studies that led to MCV licensure, MCV indications for use and labeling, with emphasis on WHO PQ MR vaccines, regulatory guidance documents (African Vaccine Regulatory Forum (AVAREF), Central Drugs Standard Control Organization (CDSCO), Drugs Controller General of India (DCGI), European Medicines Agency (EMA), United States Food and Drug Administration (USFDA), WHO) and MR-MAP-related research and strategic documents.

In parallel to the desk-based research review, experts in the fields of measles and rubella vaccination, vaccine product development, and in the development of MR microarray patches, were interviewed on the potential design of a Phase III trial, regulatory licensure strategies, and potential programmatic introduction strategies.

For the desk review and the interviews, the information sources were selected based on their relevance, completeness, and quality to identify data to inform regulatory and policy decisions for MR-MAPs. The review focused on peer-reviewed literature published in reputable journals and incorporated positions, strategies, and guidance from leading international organizations. Additionally, interviews were conducted with top experts in measles, rubella, vaccination, and microarray patches to ensure the accuracy and breadth of data. Although a comprehensive desk review was performed, it is acknowledged that this method could have been strengthened by conducting a systematic review. However, given the limited data published on MR-MAPs, the available information was likely already familiar to the authors, who are experts in the field. Factors beyond the scope of informing regulatory and policy decisions were not included in the review.

### 2.2. MR-MAP Global Convening

During 16–17 April 2024, a global convening was held in New Delhi, India by the WHO Immunization Vaccines and Biologicals Department, WHO India Country Office, and WHO South-East Asia Regional Office. The convening brought together experts and researchers in measles and rubella vaccines and microarray patches; regulatory representatives from the European Medicines Agency (EMA), United States Food and Drug Administration (USFDA), African Vaccine Regulatory Forum (AVAREF), and Drug Controller General of India (DCGI); global experts including from UNICEF, Gavi, PATH, Médecins Sans Frontières, US Centers for Disease Control and Prevention, and Coalition for Epidemic Preparedness Innovations; funders including the Bill & Melinda Gates Foundation and the RIGHT Foundation; vaccine manufacturers; MAP developers; representatives from the Indian Council of Medical Research; and the WHO HQ, Regional (WPRO, SEARO, AFRO, EMRO), and India Country office. The data obtained from desk-based research and stakeholder interviews were presented at the global convening and discussed in six breakout groups focused on (1) Phase III trial design and appropriate primary endpoint(s); (2) appropriate non-inferiority (NI) margin; (3) priority target populations; (4) concomitant vaccine delivery; (5) manufacturing strategy and shelf life; and (6) evaluation of immune responses. The recommendations reported here were formulated based on the presentations and discussions of the stakeholders and finalized during a closed meeting of the WHO Technical Advisory Group on MR-MAPs and the WHO Secretariat, which was held in April 2024.

## 3. Phase III Trial Framework—Recommendations

This framework is structured to include key elements required to inform the protocol design of a pivotal Phase III clinical trial for MR-MAPs. The national regulatory authorities (NRAs) and the WHO PQ team will need to define key aspects of a proposed study protocol. The data collected during the pivotal Phase III clinical trial should fulfill the regulatory and WHO PQ requirements and, to the extent feasible, generate evidence that is anticipated to inform a policy decision. The additional policy-related data and evidence that are expected to be needed for an MR-MAP, beyond the clinical outcomes and the chemistry, manufacturing and controls (CMC) data required for regulatory marketing authorization approval, will be described in future guidance in the form of WHO Evidence Considerations for Vaccine Policy (ECVP) [30]. The Phase III clinical trial framework will also inform the development of implementation plans for priority use cases of MR-MAPs. Such an approach, along with potential Phase IV “post-marketing” studies aimed at updating the product label or informing policy decisions (such as use in special populations or co-administration), could accelerate the pathway to programmatic introduction, uptake, and public health impact.

The following Phase III clinical trial framework considers the appropriate comparator arms within the trial design (Table 2) and considerations for non-inferiority (NI) margin (Table 3 and Table 4) for the selected primary endpoint, both of which are critical components that inform the clinical trial study arms, number of participants, and complexity of a clinical trial. The priority populations in which the immunogenicity and safety of MR-MAPs should be evaluated are discussed in Table 5. In Table 6, the administration of non-MR vaccines and other health interventions is discussed. The manufacturing considerations that could impact the design of a Phase III clinical trial are discussed in Table 7. Lastly, the appropriateness and selection of immune assays to be included in a clinical trial are covered in Table 8.

## 4. Additional Recommendations to Support the MR-MAP Pathway to Policy Recommendation and Programmatic Use

Additional data and evidence were identified during the stakeholder discussions on the pivotal Phase III trial design. Such evidence would inform and then expand policy recommendations and support programmatic implementation post initial regulatory licensure and policy.

### 4.1. Other Studies (Descriptive/Post-Market/Implementation)

Given that at least initially, MR-MAPs are expected to be used in outreach delivery and supplementary immunization activities, data pertaining to older ages, beyond 15–18 months, are likely to be required for licensure and policy decisions. Upon agreement with NRAs and WHO PQ, descriptive analyses of safety and immunogenicity in children up to five years of age could be sufficient to inform MR-MAP use in outreach and campaigns for this age range and older and could be added to the product label. After product licensure, safety and immunogenicity data could also be collected in infants younger than nine months or to evaluate interference with concomitantly delivered vaccines (with emphasis on live vaccines such as YF, polio, and JE). As such, MR-MAP developers and global health stakeholders should consider conducting additional informative clinical studies outside of the pivotal Phase III trial. In addition, if moderately to severely malnourished and HIV+ children are excluded from the trial, data demonstrating safety and immunogenicity of MR-MAPs should be collected in parallel research studies to inform a policy decision.

From a programmatic implementation perspective, it is vital to engage with Gavi-supported, self-procuring countries, other countries utilizing MRV, or those interested in the use of MR-MAP early to design and conduct implementation studies (both pre- and post-licensure) to evaluate MR-MAP’s ability to reach zero-dose children and reach other programmatic benefits, key elements of the value proposition. Prior to the Phase III pivotal trial, it is recommended to conduct research studies to evaluate that the anatomical application site or sites are safe, immunogenic, have suitable biomechanical properties to enable efficient MAP delivery, and are acceptable by health workers and caregivers. The MR-MAP application sites could be the wrist, anterolateral thigh, and/or the deltoid, for example. However, this must be confirmed in prior studies. Implementation research in parallel to the Phase III trial is required to provide program design learnings, prospective impact assessment, and other evidence required to inform integration of MR-MAPs into countries’ immunization programs. The completed, ongoing, and planned evaluations of the switch to 5-dose MRV will be an important reference for planning such research for MR-MAPs [82,83,84]. The WHO is leading the work in identifying and prioritizing implementation research questions, which will further enrich the existing knowledge and prepare for programmatic introduction [85].

### 4.2. Manufacturing, Regulatory and Policy Considerations

Defining the MAHs will be critical to advancing the timeline to regulatory submission, licensure, and commercial manufacture to generate supply for programmatic introduction. The MAH should engage with regulatory authorities and policymakers to align the regulatory and clinical strategies. The MR-MAP is a novel vaccine product, with its unique biologic-device combination configuration, and there are no existing specific guidelines from regulators or from the WHO, which is an important implication for a regulatory pathway.

The WHO Technical Report Series (TRS) includes guidelines about vaccine quality assurance and inform WHO prequalification of vaccines [24], which is required for the use of vaccines in LMICs, financed by Gavi, or procured by UNICEF. There are two WHO TRS documents relevant to the MR-MAP: TRS 840 [22] and TRS 1004 [23]. TRS 840’s manufacturing requirements sections for measles (pages 105–121) and rubella (pages 137–152) may need to be edited to accommodate the different manufacturing processes and platforms for MR-MAPs. Ideally, such revision would occur before manufacturing of Phase III clinical material, to ensure compliance with proposed standards for both the drug product and the device components, as Phase III clinical material are expected to be representative of initial launch material.

Another consideration is the WHO programmatic suitability criteria for the PQ of the vaccine [31], which were developed taking into account vial presentation and syringe reconstitution and delivery. MR-MAP vaccines should meet the mandatory criteria; however, a revision to this document may be valuable in order to take into account specific aspects of MR-MAP devices [31].

The NRAs targeted for initial licensure and for Phase III conduct will need to opine on the overall design of the pivotal Phase III trial, including the critical questions of immunologic endpoints and their NI margins. Those areas not addressed in the Phase III design could be included in additional Phase III studies or research post-licensure as noted above. Discussions with NRAs and WHO PQ need to include aspects related to manufacturing consistency, specifications for lot release, and end-of-shelf life (EOSL) potency. Given that current MR vaccines are primarily produced in India, the CDSCO will likely play a critical role in the regulatory licensure of MR-MAP products. Importantly, if the Phase III trial is conducted in India, a Phase II leading arm might be considered for addition to the trial to address the need for a Phase II/III bridging trial with Indian infants and children for licensure of vaccines imported or from technology transfer (as opposed to domestic development).

After regulatory licensure and pending WHO PQ, MR-MAPs will require a policy recommendation from global or country stakeholders to allow for MR-MAP introduction and use in countries. The WHO, informed by the Strategic Advisory Group of Experts on Immunization (SAGE), can issue a policy decision on MR-MAPs, allowing for their financing by Gavi and procurement by UNICEF. To determine appropriate data to inform policy, SAGE considers the evidence-to-recommendations tables [29] and the ECVP [30]. Data showing MAP impact and cost-effectiveness modeling will likely be required for the policy. However, it is recognized that increased coverage cannot be demonstrated before MAPs are licensed and deployed. Data showing the acceptability and feasibility of health workers’ use of MAPs, acceptability in zero-dose populations, other programmatic benefits, and ability to reach remote populations, together with modeling, could be used to inform policy decisions.

## 5. Conclusions

It is anticipated that MR-MAPs will play a vital role in immunization programs, particularly in reaching zero-dose children through outreach and campaign efforts, addressing the coverage gap for MRVs, and contributing to MR elimination. The proposed Phase III trial framework attempts to align regulatory, policy, and immunization program needs and is intended to address the recommendations from the MR-MAP TAG, other key stakeholders, regulatory, and WHO PQ and policy requirements. The proposed framework has also been developed to anticipate and prevent potential challenges associated with the design and running of a clinical trial, particularly in LMICs, such as inclusion of appropriate populations, or ensuring that the non-inferiority margin is appropriately defined and measured. This manuscript focuses on aspects related to the design of the Phase III clinical trial for MR-MAPs and other important considerations common to all vaccine trials, such as regulatory and ethical consultations, site selection, staff training, participant recruitment, and logistics, are addressed through adherence to international guidelines, and not discussed here [86].

The pivotal Phase III trial, which should be an individually randomized (1:1), open-label NI trial to receive an MR-MAP or MR-SC at 9–10 months of age to assess safety and non-inferiority of immunogenicity of MR vaccines delivered by a MAP, six weeks after the first dose of the MR vaccine. Dependent upon the NRA and the WHO PQ team review and agreement of the proposed pivotal Phase III trial framework, the trial could continue in a subset of children who would be randomized to receive either the same vaccine presentation or a different vaccine presentation 6 months after the first MR dose, at 15–16 months of age. The trial should be conducted in countries where MR-MAPs are to be utilized programmatically and in representative populations regarding health status, informed by the use cases and projected demand for the product in LMICs.

The relevant NRAs and WHO PQ should confirm the acceptability of the proposed immunogenicity endpoints and their NI margin as part of the overall clinical data set that will be submitted for regulatory approval and should opine on the overall Phase III trial design. Consultations involving both NRAs and the WHO PQ team concurrently should be favored to minimize delays to Phase III start.

In addition to the pivotal Phase III trial, additional clinical studies may be required to meet regulatory, WHO or programmatic needs for MR-MAPs. Such studies could address different age groups outside of the 9 to 16-month-old age group, such as younger infants (down to 6 months old) or older children and adults. The studies could also address the safety and immunogenicity of MR-MAPs in moderately-to-severely malnourished children, should they be excluded from the Phase III trial. Concomitant vaccine administration, with priority live virus vaccines that may present the potential for interference, may also need to be addressed. Such studies may be possible pre- or post-licensure, depending upon the final clinical and regulatory strategy.

Determination of the MAH and Phase III trial sponsor will be vital to advancing MR-MAPs through the regulatory licensure process and WHO PQ, and eventual commercialization, ensuring sufficient supply for programmatic introduction. Ideally, this responsibility should be taken on by a manufacturer of prequalified MR vaccines. The NRAs for initial licensure and WHO PQ team will need to address outstanding questions regarding regulatory requirements for quality aspects (manufacturing, controls) of the biological and the device components of MR-MAPs.

Pathways to facilitate joint NRA and WHO PQ reviews such as the EMA M4-all should be explored to determine alignment with an MR-MAP regulatory and marketing strategy. However, before implementing a pivotal Phase III trial, completing MR-MAP device design development will be critical to address issues such as human factors, application site(s), wear time, and cGMP compliant manufacturing facilities. Validation of manufacturing and controls in such facilities is required to produce the Phase III clinical product and prepare for the regulatory licensure process, WHO PQ, and meeting the initial product demand once it is ready for immunization program use. From a WHO policy perspective, the development of the WHO ECVP should be advanced to identify data and evidence to support policy needs, building upon this Phase III framework that describes the parameters for a pivotal licensure trial.

## Figures and Tables

**Figure 1:**
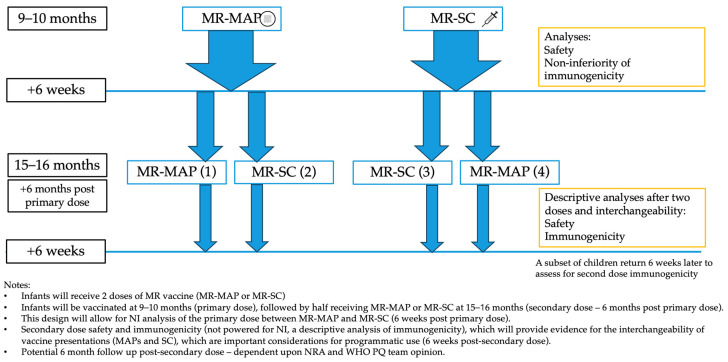
Proposed Phase III Trial Design.

**Figure 2:**
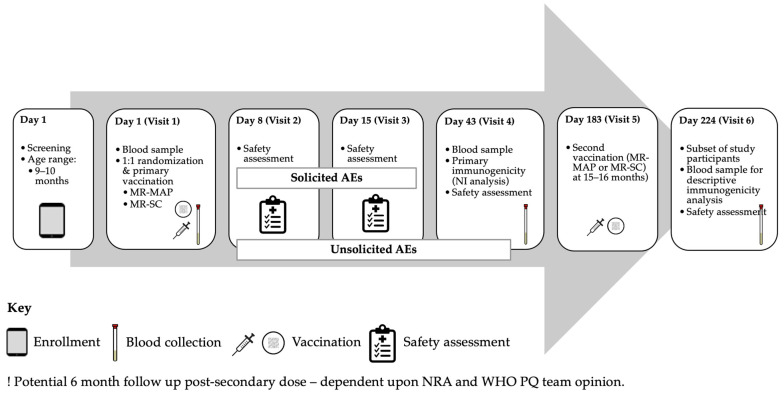
Illustrative Phase III Trial Flowchart.

**Table 1:** Desk-based Review Documents.

Global Guidelines	Summary
WHO Position papers on Measles and Rubella [3,4]	Provide the WHO’s official recommendations and guidance on measles and rubella vaccines, including vaccination schedules, target groups, and strategies for global immunization efforts.
WHO/UNICEF MR-MAP Target Product Profile [10]	Defines the desired characteristics of MR-MAPs, outlining the requirements for product development to meet global public health needs.
PDVAC MR-MAP meeting report [21]	Summarizes the discussions and outcomes from a meeting of the WHO’s Product Development for Vaccines Advisory Committee (PDVAC) regarding the development of MR-MAPs
WHO Technical Report Series for MMR, clinical trials and prequalification requirements [22,23,24,25,26]	Technical guidelines and standards issued by the WHO covering the clinical trials, manufacturing, and prequalification criteria for measles, mumps, and rubella (MMR) vaccines.
WHO prequalified MR vaccine package inserts [27,28]	Official package inserts for measles–rubella vaccines that have been prequalified by the WHO, detailing the vaccine’s composition, usage, and safety information.
WHO strategy, policy and programmatic requirements [29,30]	Documents detailing the methods and processes used by the WHO’s Strategic Advisory Group of Experts on Immunization (SAGE) to develop evidence-based immunization policies, along with a generic framework for considering evidence in vaccine and monoclonal antibody policy development.
WHO PQ and product testing [31,32,33,34]	Guidelines and procedures for the WHO prequalification of vaccines, including testing methods and quality assurance processes.
**National guidelines**	
National regulatory authority guidance documents for clinical trials and non-inferiority margin [35,36,37,38,39,40,41,42,43,44,45,46,47,48]	Guidelines from various national regulatory authorities on conducting clinical trials, with a focus on determining non-inferiority margins in vaccine studies.
**Research studies**	
MR-MAP published research, study protocols and strategic documents [9,14,18,19,49,50]	Collection of research papers, study protocols, and strategic documents focused on the development, testing, and implementation of MR-MAP technology.
Alternative delivery technology published research [51,52,53,54,55,56,57,58,59,60,61,62,63]	Compilation of research on various innovative vaccine delivery technologies beyond traditional injection methods, including needle-free devices and other novel approaches.
Measles containing vaccine research, including studies that led to regulatory licensure or WHO PQ [64,65,66,67,68]	Research studies and clinical trials that have contributed to the licensure and WHO prequalification of measles-containing vaccines.

**Table 2:** Clinical Trial Design.

The proposed design for a pivotal Phase III clinical trial includes 9–10 month-old MR naïve infants, immunized with two doses of MR SC or/and MR-MAP vaccines, administered six months apart, to assess immunogenicity and safety after the first dose, and provide a descriptive analysis of safety, immunogenicity, and interchangeability of vaccine presentations (MAP then N&S, or the converse) after the second dose of an MR vaccine. **Question** Is the proposed design appropriate for a pivotal Phase III clinical trial?
**Discussion** The WHO position paper on measles notes that a single dose of the MCV will provide long-term protection for healthy vaccinees, with measles virus antibodies detected throughout the life of the individual. Research indicates that waning immunity may be a contributing factor in some cases of secondary vaccination failures; however, it does not seem to have a significant epidemiological impact on the transmission of the measles virus. The WHO recommends two doses for primary immunization and notes that measles vaccines are safe and effective, and vaccines from different suppliers may be used interchangeably within immunization programs [3]. Based upon this information, some regulatory authorities and WHO SAGE may require safety and immunogenicity data for two doses of the MR-MAP, including demonstrating interchangeability with the existing MCV and an MR-MAP to inform consideration of a policy recommendation. Measurement of immunogenicity after the last dose of a vaccination series is noted as the standard approach, be it the evaluation of seroresponse rate (SRR) or a geometric mean concentration (GMC) of neutralizing antibodies (see Table 3 for further information). However, if primary vaccination elicits immune memory in naïve vaccinees, post-primary doses may not allow for detecting differences and may be more appropriate as a secondary endpoint [37]. Given that a single dose of the MCV is highly immunogenic with seroprotection rates consistently above 90% in >9 M old infants, the primary endpoint for a pivotal Phase III trial of MR-MAPs could be assessed six weeks after the first dose of an MR vaccine. A six-week follow-up after receiving the second dose of the MR vaccine (either MR-MAP or MR-SC) should be sufficient to assess immunogenicity in a subset of trial participants. Per the WHO guideline on clinical evaluation of vaccines, reports of SAEs should be collected for all trial participants 6 months post final dosing [23]. The trial sponsor and/or market authorization holder (MAH) should confirm the adequacy of this approach as part of pre-Phase III consultations with regulatory authorities. The evaluation of the relative persistence of antibody responses one or two years after one or two vaccinations, however, could be conducted either in a sub-study to the pivotal Phase III trial or in a separate study. This will be considered after the MR-MAP is licensed and utilized programmatically.
**Recommendations** A primary consideration should be given to a pivotal Phase III trial (Figure 1), which would be an individually randomized (1:1), open-label NI trial to receive an MR-MAP or MR-SC at 9–10 months of age (measles–rubella naïve infants) to assess non-inferiority of immunogenicity and safety (primary endpoints) of MR vaccines delivered by a MAP. The primary NI analysis of safety and immunogenicity of the MR-MAP could be conducted at six weeks after the first dose of the MR vaccine [19,68]. As a secondary consideration, should regulatory agencies and policy makers require, the trial should continue in a subset of children who would be randomized to receive either the same vaccine presentation or a different vaccine presentation (those who received an MR-MAP would receive MR-SC and vice versa) 6 months after the first MR dose, at 15–16 months of age. Consequently, the trial would include four arms: primary and secondary doses of an MR-MAP (arm 1); primary dose of an MR-MAP followed by a secondary dose of an MR-SC (arm 2); primary and secondary doses of an MR-SC (arm 3); and primary dose of an MR-SC followed by a secondary dose of an MR-MAP (arm 4)–see Figure 1. Data from children who were vaccinated with the second dose of the MR vaccine at 15–16 months old could be descriptively evaluated six weeks after the second dose of the MR vaccine to assess the immunogenicity and safety of using two MR-MAP doses, as well as using two MR vaccine presentations (MAP and SC) interchangeably. However, the sample size of a trial needs not be powered for a formal NI analysis of immunogenicity after the second dose of an MR vaccine (see Figure 1 and Figure 2). To address potential ethical concerns, all trial subjects should receive two doses of the MR vaccine (MR-MAP or MR-SC) to complete their vaccination series, as well as other co-administered vaccines, depending upon local immunization program requirements. Safety would be assessed immediately after each vaccination and likely for all vaccinated infants, and the extent of the safety endpoint data required is dependent upon NRA and WHO PQ team agreement. The number, severity, and relatedness of solicited local and systemic adverse events (AEs) should be collected on the day of MR vaccine administration and daily until two weeks post-vaccination, as per the Division of AIDS (DAIDS) Table for Grading the Severity of Adult and Pediatric Adverse Events [69]. Skin discoloration is an expected event in dark-skinned individuals and should be specifically assessed [19]. The number, severity, and relatedness of all unsolicited (AE) should be assessed six weeks after each vaccination, and unsolicited serious adverse events (SAEs) for the duration of the clinical trial (potentially up to 6 months post-second dose for SAEs dependent upon NRA and the WHO PQ team opinion). Prior to the Phase III pivotal trial, it is recommended that the anatomical application site or sites be determined to be safe, immunogenic, have suitable biomechanical properties to enable efficient MAP delivery, and must be acceptable by health workers and caregivers. The MR-MAP application sites could be the wrist, anterolateral thigh, and/or the deltoid, for example; however, this must be confirmed in prior studies. Optimally, the wear time would be under one minute. The leading MR-MAP developers are working to determine the wear time in upcoming research studies. The NRA(s) considered for initial marketing authorization application will need to confirm if the proposed trial design would be acceptable for licensure and, therefore, should be consulted as part of pre-Phase III regulatory consultations. Justification for and nature of a descriptive analysis post-secondary dose will need to be presented by the trial sponsor to the NRA.

**Table 3:** Non-inferiority margin for seroresponse rates comparison.

When designing a vaccine clinical trial, determining an appropriate NI margin is crucial for ensuring the trial’s validity and reliability. The non-inferiority margin defines the acceptable difference in immunogenicity or efficacy between the new vaccine and the established reference vaccine, indicating that the new vaccine is not significantly worse than the reference within a pre-specified limit. Selecting this margin impacts several key aspects of the trial, including the sample size needed to achieve the required statistical power and the likelihood of trial success or failure. A narrower margin always demands a larger trial to achieve sufficient power, increasing costs and complexity, while a wider margin may raise concerns about the use of a less immunogenic or efficacious vaccine. Moreover, the choice of NI margin can directly influence the probability of the new vaccine’s introduction into the market, as it determines the threshold at which the vaccine is deemed effective enough for licensure and widespread use. This discussion will explore these factors and their implications for vaccine approval and public health. **Questions** Given the safety and effectiveness of the current MR vaccines, should the commonly used margins of 5 or 10% be used to demonstrate non-inferiority of immunogenicity between the two vaccine presentations? What are the considerations for the non-inferiority margin from a policy perspective?
**Discussion** There are two assessments to consider in selecting primary endpoints to assess immunogenicity NI for MR-MAPs relative to MR-SC: seroresponse rate (SRR) difference and Geometric Mean Concentration (GMC) ratio. The SRR difference relates to the proportion of trial subjects in which a minimum protective antibody level is induced six weeks after vaccination (for measles ≥ 200 mIU/mL, for rubella ≥ 10 IU/mL ref). The GMC ratio is the geometric mean magnitude of the immune response relative to the comparator (MR-MAP compared to MR-SC). A published meta-analysis of any vaccine NI randomized clinical trials indicated that most NI margins were 10%, although some vaccine NI studies had a lower NI margin such as 5% [70]. Based on such analysis, the study identified general considerations for selection of NI margins [70]. MCV products have been licensed and prequalified by the WHO based on clinical trials with a 5 and 10% NI margin difference in SRR [28,52,66,67,68]. There are advantages and disadvantages for each of the NI margins, which are presented in Table 4. As per the US FDA, the NI margin is usually determined by what would be clinically acceptable in terms of loss of effect [43]. The agency notes that planning for an NI study tends to be conservative, often confirmed by ensuring that the upper bound of the 95% confidence interval is less than the NI margin. Per the EMA, guidance on the NI margin should be sought from the Committee for Medicinal Products for Human Use (CHMP) through scientific advice. Mortality rate and disease risk are clinical considerations that should be referenced when determining the NI margin, and it was noted that sample size should not be a consideration [36,43]. Per the CDSCO, pivotal clinical trials should use a predetermined and justifiable NI margin. The determination of a NI margin should consider the clinical significance of the endpoint, the severity of the disease, target population susceptibility, the availability of an established immune correlate of protection (ICP), and the assay performance characteristics. A more stringent NI margin may be suitable when the vaccine aims to prevent severe or life-threatening diseases and/or will be used in particularly vulnerable populations, such as infants. The use of a stricter NI margin might be suitable in cases where there is a chance of reduced immunogenicity, such as when comparing a new vaccine only with those approved based on NI trials. On the other hand, if a new vaccine is recognized to provide substantial safety advantages or better coverage, the agency states that less strict NI margins could be considered [41].
**Recommendations** As international standards for defining measles and rubella seroprotection exist, the primary endpoint should be NI of the SRR (MR-MAP relative to MR-SC) [3,4]. The NI analysis can be conducted six weeks after receipt of the first dose of an MR vaccine. Should all children be vaccinated with two doses, the NI analysis can be conducted six weeks after receiving the second dose of the MR vaccine. A comparison of an incremental increase in immunogenicity between the first and second vaccine dose would be challenging given the expected marginal increases in the proportion of seroconverted individuals compared to when measured six weeks after the first dose of an MR vaccine. Analyses of immunogenicity and safety after the second dose of an MR vaccine could be a secondary endpoint based on descriptive statistics. Figure 2 illustrates an example of a trial protocol. Ultimately, the appropriateness of the proposed NI analysis should be confirmed with the relevant NRAs during end-of-Phase II/pre-Phase III consultations.

**Table 4:** NI Margin—Advantages and Disadvantages.

Non-Inferiority Margin	Advantages	Disadvantages
**5%**	High immunogenicity with minimum or no inferiority to current vaccines is desired, given the high transmission of measles and need for a high proportion of seroprotected individuals. Highly effective vaccines exist, are routinely implemented and are an important reference point for countries—generating data at 5% NI will provide greater confidence to countries to introduce MR-MAPs into programmatic use. A WHO Listed Authority (WLA) may require a 5% NI for the pivotal Phase III trial [71].	Very stringent success criterion for a Phase III trial—failing to meet the NI threshold, even marginally, may delay and/or prevent the technology from moving forward into programmatic use and reaching public health impact. If potency variability of released MR-MAPs or MR-SC products exists, this may result in variations in immunogenicity and increase the potential for the product to fail at 5% NI.
**10%**	Reduces costs and accelerates potential time to product licensure, while meeting select NRA recommendations that led to MCV product licensure and WHO PQ. The potential decrease in immunogenicity could be offset by an increase in vaccine coverage, with important public health implications such as reaching zero-dose children. Published results comparing MCVs (single dose) with 10% NI margin led to successful product licensure and use. A 10% non-inferiority margin could accommodate greater variability in immunogenicity within the trial, which, in turn, may allow for the inclusion of broader and more diverse populations (HIV+, malnourished children, broad age range 9–12 months), increasing the generalizability of the trial results to a wider population.	A highly effective vaccine exists—there is the potential that as a novel delivery technology MR-MAPs may be perceived as a less effective and will negatively impact acceptability, product uptake and use in countries. The use of MR-MAPs may fail to translate to increased vaccine coverage, and coupled with decreased immunogenicity it may lead to increased MR incidence.

**Table 5:** Vaccinated Populations.

In the context of clinical trials, the inclusion of additional populations, such as those with underlying health conditions or diverse demographic groups, is crucial for ensuring that the trial’s findings are applicable to real-world scenarios. Ideally, data collected from a trial should support the expected use of a vaccine in various contexts, such as during public health campaigns or outbreak responses. However, achieving this ideal can be challenging due to pragmatic considerations, such as limitations in resources, ethical concerns, and logistical constraints. Expanding the trial population to include groups like HIV-positive individuals, malnourished children, or broader age ranges can enhance the generalizability of the results and provide valuable insights into the vaccine’s efficacy and safety across different subgroups. This discussion will explore the key factors that must be considered when deciding whether to include additional populations in the pivotal Phase III trial for MR-MAPs. **Questions** In addition to the 9–10-, and 15–16-month-old children, what (if any) other age groups or priority populations should be included in the Phase III trial and required for a policy decision? Should malnourished and HIV+ children be included in the Phase III trial? What is the minimum level of country, population, and regional diversity needed to be considered in the Phase III trial and required for policy decision?
**Discussion** From a Phase III trial implementation perspective, the enrollment of 9–10-month-old infants for the primary dose (and not 9–12 month olds), who six months later at age 15–16 months (and not 15–18 months) would receive their second dose in alignment with the current schedule, will allow for a more focused implementation of the trial, marginal expected differences in immunogenicity, and higher chance of reaching the NI margin while broadly supporting use in children less than two years of age. In addition to routine immunization, MCVs are used in supplementary immunization activities, measles outbreak response, and other delivery strategies, often extending the vaccine use to older populations [3,72]. As such, additional data indicating safety and immunogenicity in older children is likely to be required to inform regulatory and policy decisions. This could be achieved in pre- or post-licensure studies. PDVAC has recommended that moderately-to-severely malnourished infants should be included in the pivotal clinical trial; however, their inclusion in a pivotal licensure trial presents a challenge to the trial, as malnourished children, especially those with vitamin A deficiency, have an increased risk of developing severe or fatal measles [21]. Using stratified randomization to allocate malnourished infants equally to the MR-MAPs or MR-SC trial arms will minimize trial bias when evaluating NI. PDVAC has recommended that infants living with HIV should be included in the clinical development, either during Phase II, III, or in additional studies that run parallel to Phases II–III. The limited evidence available indicates that measles vaccines are safe in HIV-infected children who are not severely immunocompromised, and measles vaccines are recommended for use in that population [3,21]. The immunogenicity of MCVs in children living with HIV and on antiretroviral therapy appears to be similar to the immunogenicity in children without HIV [73,74]. MR-MAPs could play an important role in the response to measles outbreaks, including in infants younger than 9 months. However, the inclusion of 6-month-old infants in a pivotal Phase III trial risks extending the timeline and increasing the costs and complexity of the trial. In countries with high incidence of measles and HIV infection, a zero dose of measles at 6 months (MCV0) is recommended [3]. This recommendation will need to be considered in the context of enrolling measles–rubella vaccine (MRV) naïve infants in the pivotal Phase III trial and selecting the countries to conduct the trial.
**Recommendations** In the pivotal Phase III trial, the decision to include moderately to severely malnourished or HIV+ infants who are on anti-retroviral therapy should consider the incidence of malnutrition and HIV at a site of the trial, the NI margin (a higher margin is more permissive of differences in immunogenicity between vaccines), logistical and operational challenges associated with recruiting and retaining HIV+ and malnourished infants in the trial, including coordinating with HIV and nutritional treatment regimens and programs, and implications of including these populations on the generalizability of the trial results. If moderately to severely malnourished or HIV+ infants are included in a trial, they should be evenly assigned to treatments by stratified randomization to either the MR-MAP or MR-SC trial arms, to ensure balanced enrollment. Their data can be part of the full analysis set; however, they may be excluded from the per-protocol analysis, meaning the data would not be part of the analysis to determine comparability in immunogenicity and safety between the MR-MAP or MR-SC. There is a risk of increasing the heterogeneity of the immune response and the chances of not meeting the NI margin. Although these infants may comprise a small proportion of the trial’s participants, their responses are likely to be carefully assessed by NRAs and the WHO PQ team due to their vulnerable status. Should the moderately-to-severely malnourished or HIV+ infants not be included in the Phase III trial, a separate study should be conducted to collect data on safety and immunogenicity of MR-MAPs, which may be required to inform a policy decision. 6-month-old infants should not be included in the Phase III trial but rather be evaluated post-licensure to simplify the pivotal Phase III trial. Data describing immunogenicity and safety in older children up to 5 years old should be evaluated in a separate descriptive trial, ideally pre-licensure upon consultation with relevant NRAs. Such data are likely to be required for a policy decision and sufficient to inform the use of MR-MAPs in children older than 5 years old and adults. The trial should be conducted in multiple countries that appropriately represent those populations where MR-MAPs will be used, accounting for HIV status, malnutrition, and other factors that may influence the safety/reactogenicity and immunogenicity of MR-MAPs.

**Table 6:** Concomitant delivery of vaccines, vitamins, and micronutrients.

Measuring the concomitant delivery of vaccines in clinical trials is crucial for ensuring the safety and immunogenicity of vaccines and the effectiveness of immunization programs. When vaccines are administered together, there is potential for interactions that could impact their vaccine’s immunogenicity, effectiveness, or safety profiles. Understanding these interactions is essential to confirm that co-administration does not compromise the immune response or increase the risk of adverse effects. **Questions** Should data demonstrating the safety and immunogenicity of the MR-MAP delivered concomitantly with other vaccines and supplementation with vitamins and micronutrients that are typically administered at the 9–12 month and 15–18-month timepoints be collected in the Phase III trial? Which vaccines should be considered and why? Are data demonstrating safety and immunogenicity of the MR-MAP delivered concomitantly with other vaccines (“co-administration”) required to inform the policy decision? If yes, what are the recommended vaccines that should be considered?
**Discussion** The standard approach is to design dedicated NI trials and confirm non-interference for novel vaccines targeted for infant use by comparing co-administration versus staggered administration of the new vaccine with licensed vaccines given at the same age [23]. However, the pivotal Phase III trial may not need to be powered to evaluate interference with concomitantly delivered vaccines, vitamins and micronutrients, and inclusion of additional trial arms to evaluate the impact of concomitant vaccines will significantly complicate and increase the cost of the Phase III trial. Live vaccines should be delivered concurrently with MCVs or at 4-week intervals. The oral poliovirus vaccine (OPV) is one exception, which can be delivered without interference before, at the same time, or after measles immunization [3]. For delivery of the MCV aligned with the EPI schedule (9–12 and 15–18 months), some vaccines need to be delivered concomitantly and are standard of care, which are region- and context-specific: Yellow Fever Vaccine (YFV), Meningococcal (A, C, Y, W, X) Polysaccharide Conjugate Vaccine (MenFive™), polio (IPV/bOPV2), Typhoid Conjugate Vaccine (TCV), Malaria, Diphtheria-Tetanus-Pertussis, Hepatitis A, Japanese Encephalitis (JE), and Pneumococcal Conjugate Vaccine (PCV). Notably, MMR and YF vaccine co-administration may result in interference in children under 2 years of age [3]. Concomitant delivery of the RCV can occur with inactivated and live virus vaccines; the RCV should be delivered concurrently with the MCV. In studies of co-administration of the RCV with the YF vaccine, lower GMCs for both antigens were observed; however, the values were well above the threshold necessary for seroconversion [4]. The WHO recommends co-administration of the RCV and the YFV despite the potential lower titers of rubella and YF antibodies. The rationale for this recommendation is that the potential reduction in immune response (lower titers) is outweighed by programmatic implications of delaying vaccinations, and the potential for a more significant impact on population immunity with co-administration [4]. Vitamin A, Vitamin D, and iron supplements could be provided to infants as needed in the Phase III trial per WHO guidelines and country health status (high rates of Vitamin A deficiency, malnutrition, anemia) and local programmatic practice [75,76].
**Recommendations** If other vaccines are given at the same age as MR in an immunization program setting, then during the Phase III trial such vaccines could be given concomitantly or within one month, dependent upon consultation with NRA and the WHO PQ team. The immune response of other vaccines could potentially be evaluated, and comparisons made between MAP and N&S SC groups using the same co-administered vaccines for dose 1 and then for dose 2 (separate comparisons). Data showing concomitant delivery of live vaccines such as YF, JE, or polio are likely to be required due to potential for interference. The delivery of dietary supplements should also be considered when designing the pivotal Phase III trial. Alternatively, such data could be collected through other studies completed prior to regulatory and policy review [77]. The inclusion of concomitant vaccines in the Phase III trial will require discussions with relevant NRAs and the WHO PQ team to obtain agreement.

**Table 7:** Manufacturing and Shelf Life.

It is essential to evaluate manufacturing consistency before licensure as a critical aspect of vaccine product development and manufacturing in the initial launch facilities. For MCV product lot release, virus concentration and thermostability per the specified shelf life must be assessed [22]. The assessment of consistency should be conducted on successive lots [25]. Such an approach allows for the assessment of any potential differences in immunogenicity between MCVs at the beginning and end of their shelf life, ensuring that the vaccine remains effective throughout its intended use period. This approach helps to identify any potential discrepancies or variations that could affect the overall effectiveness and safety of MCVs when administered to the broader population. **Questions:** Should a comparison of immunogenicity between MR-MAPs lots (lot-to-lot (L2L)) be included in the pivotal Phase III trial? If so, how would that impact the design of the trial? Will it be required to generate safety data for a high-dose MR-MAP (maximum possible production dose, given the variations in MAP delivery at different points of MAP shelf-life and the associated dose)?
**Discussion** The WHO recommends that assays to identify and determine viral concentrations should be used for MCVs. Such assays are required due to the potential impact of diluents and stabilizers on the potency and stability of the individual antigens. Given that the MR vaccine given by a MAP will have a modified formulation to MR-SC, such tests will need to be conducted for the measles and for the rubella vaccine that are given via the MAP [22]. For each vaccine, live virus concentration of the individual antigens for an individual lot should be determined by cell culture during vaccine manufacturing. For both measles and rubella, a minimum of 1000 viral infective units measured by a 50% cell culture infectious dose (CCID50) has been demonstrated to be immunogenic in MR SC trials. “At least three” containers (such as individual vials) randomly selected would be assessed individually from at least three consecutive Drug Product production lots. The geometric mean infectious virus titer of each container should meet the minimum requirements after incubation, with the virus titer decrease not exceeding 1.0 log. Thermostability testing should also occur with the selected containers at 37 °C for seven days [22]. Another consideration related to the thermostability testing described in Technical Report Series (TRS) No. 840 is the MR-MAP TPP recommendation [10] to conduct testing for VVM 30 (37 °C for 30 days at the beginning of shelf life) [33] and CTC (40 °C for three days at the end of shelf life) for product labeling [34]. The formulations being tested in pivotal trials are typically manufactured using validated processes and go through lot release per NRA and WHO TRS requirements just like they would for the commercial product [23]. WHO TRS states that one batch of vaccine produced through each method might be enough for the comparison; however, data from multiple lots might be required. In vaccine development, typically, three lots are utilized when conducting lot-to-lot consistency in a clinical trial. Previous MCV trials have included at least three lots of the MCV and the estimated NI of immunogenicity as well as the reactogenicity and safety between the evaluated arms while combining the evaluated lots [78]. Some studies have also analyzed the immunogenicity between the MCV lots within the same arm; however, the trial was not powered to evaluate non-inferiority between each MCV lot [64]. The evaluation of a maximum production dose (high potency) for an MR-MAP product may not be required for the pivotal Phase III trial; however, this could be addressed in upcoming Phase II or non-pivotal Phase III studies. Similarly, the lowest acceptable levels of potency at end of shelf life may need to be evaluated in an appropriately designed trial to demonstrate acceptable clinical performance [23,79]. The NRAs for the initial marketing authorization application (MAA) and the WHO PQ team, which will likely need assurance that the proposed potency specifications for lot release, have been demonstrated to be acceptable during clinical development. These considerations should also be addressed during end-of-Phase II/pre-Phase III regulatory consultations [35].
**Recommendations** Manufacturing and analytical L2L consistency from a CMC perspective must be demonstrated prior to the pivotal Phase III trial and at the time of authorization of commercial lots. MR-MAP thermostability and potency (high and low doses related to manufacturing and product shelf life) testing should be conducted prior to the pivotal Phase III trial. To ensure representativeness, three production lots of MR-MAPs with similar expiry dates should be included in the pivotal Phase III trial. NRAs and/or the WHO PQ team may require clinical data demonstrating the comparison of equivalent immunogenicity among three production lots of MAP. If so, the sample size for the pivotal Phase III trial will need to be adjusted to assure adequate statistical power and control of the Type I error. Owing to the unique nature of the MR-MAP, including both a biological and a device component, MR-MAP developers should seek guidance from NRAs including WLAs and from the WHO PQ tean regarding their proposed CMC and device data package for licensure. Such consultations should ideally be initiated after successful Phase II completion.

**Table 8:** Evaluation of Immune Responses.

Evaluation of immune responses is essential in measuring immunogenicity between different MCVs. Both humoral and cellular immune responses could be included in such evaluation; however, it is recognized that humoral responses, typically measured through assays that detect specific antibodies, play a major role in protection against measles and rubella. To ensure the accuracy and reliability of these measurements, it is imperative that the assays used to evaluate immune responses are rigorously validated. Validation ensures that these assays are precise, reproducible, and suitable for establishing the immunogenicity and non-inferiority of MCVs in clinical trials. This section will explore the recommended assays and approaches for evaluating immune responses to MCVs, drawing on established guidelines and recent advancements in the field. **Question:** What immunological and functional assays should be used to evaluate the immune response to MR-MAPs?
**Discussion** Neutralizing antibodies targeting the H antigen play a crucial role in preventing measles virus infection. However, once an individual is infected, cell-mediated immunity becomes essential for clearing the virus. Post wild-type viral infection, the presence of both circulating measles virus-specific CD4+ and CD8+ T cells, as well as the continuous production of measles virus-specific antibodies, contributes to the establishment of a long-term, potentially lifelong, immune memory. Protection from infection is sustained through the rapid development of secondary humoral and cellular immune responses, even as the levels of anti-measles virus antibodies may diminish over time [3]. For rubella, the RA27/3 strain develops antibody levels that closely mimic those generated by natural infection. During clinical trials, a single dose of the vaccine led to the development of rubella antibodies in 95–100% of susceptible individuals aged ≥9 months. A meta-analysis of 26 clinical trials revealed that 99% (95% CI, 98–99%) of children aged 9–18 months produced antibodies after receiving a single dose of the rubella vaccine containing the RA27/3 strain. Administering a second dose of the rubella vaccine with the RA27/3 strain resulted in 100% seroconversion (99–100%) [4,80]. Per the EMA, assays used for measurement of primary or secondary endpoints are required to be validated and calibrated to relevant international standards. Immune response characterization for a new vaccine can include functional and/or binding antibody determination, immune response kinetics, immune memory induction, or humoral immune response-related aspects such as previous vaccination or natural infection/exposure or cross-reactive antibody, cross-priming or correlation between cytokine/gene expression profile, or an ICP. CMI assessment is also encouraged [37]. Per the CDSCO, reference to existing immune response information related to similar, approved vaccines may be suitable for characterization of a new vaccine. The past preclinical and clinical research conducted will inform the CDSCO regarding what will be required for a Phase III trial. In the case of measles and rubella, an ICP for each has been established. Assessment of the humoral response (functional antibody and/or total antibody) after vaccination may be sufficient. Total immunoglobulin (IgG) can be suitable for clinical trials if a correlation between functional and total antibody responses exists. However, in specific age groups and target populations the functional immune response might need to be determined. Per the CDSCO, Cellular Mediated Immunity (CMI) is highlighted as a potential area of investigation for new vaccines to support research conducted on the humoral immune response and could provide useful information to compare between treatment groups [41].
**Recommendations** Serum neutralizing antibody assays (SNAs) were proposed to measure immunogenicity endpoints (measles and rubella). Measles seroprotection would be defined, based on current international standards as ≥200 mIU/mL, and rubella seroprotection would be defined as a standardized titer of ≥10 IU/mL. The acceptability of these cutoff values, as well as the appropriateness of the associated immunological assays, however, will need to be confirmed by the NRAs and the WHO PQ team. Other endpoints would include the seroresponse rate (% attaining or exceeding the minimum protective threshold) and GMCs. The microbead assay (MBA) is being validated and could be ready for the Phase III trial as a potential alternative or used in addition to the SNAs [18,19,81]. The MBA could allow for rapid immunogenicity testing for both antigens in a centralized laboratory with harmonized protocols. The regulatory authorities and the WHO PQ team would need to confirm the suitability of that assay in MR-MAP clinical development. CMI assessment could be an exploratory objective in earlier phase trials or potentially the Phase III trial (subset of samples); however, concerns were raised about increasing the complexity of the trial—which likely will not be required from a regulatory licensure perspective.

## Data Availability

The data presented in this study are available in this article.
